# Technical improvements in preparing 3D printed anatomical models for comminuted fracture preoperative planning

**DOI:** 10.1186/s41205-023-00189-5

**Published:** 2023-09-11

**Authors:** Naomi C. Paxton, Brandon G. Wilkinson, Daniel Fitzpatrick, Erin C. Owen, Simon Luposchainsky, Paul D. Dalton

**Affiliations:** 1 Phil & Penny Knight Campus for Accelerating Scientific Impact, University of Oregon, 1505 Franklin Blvd, Eugene, OR 97403 USA; 2 Slocum Center for Orthopedics and Sports Medicine, Eugene, OR USA; 3 Slocum Research & Education Foundation, Eugene, OR USA

**Keywords:** Surgical planning, 3D modelling, 3D printing, Orthopedics, Comminuted fracture

## Abstract

Preoperative planning of comminuted fracture repair using 3D printed anatomical models is enabling surgeons to visualize and simulate the fracture reduction processes before surgery. However, the preparation of such models can be challenging due to the complexity of certain fractures, particularly in preserving fine detail in bone fragments, maintaining the positioning of displaced fragments, and accurate positioning of multiple bones. This study described several key technical considerations for preparing 3D printed anatomical models for comminuted fracture preoperative planning. An optimized segmentation protocol was developed that preserves fine detail in bone fragments, resulting in a more accurate representation of the fracture. Additionally, struts were manually added to the digital model to maintain the positioning of displaced fragments after fabrication, reducing the likelihood of errors during printing or misrepresentation of fragment positioning. Magnets were also used to enable separation and visualization of accurate positioning of multiple bones, making it easier to visualize fracture components otherwise obscured by the anatomy. Finally, the infill for non-target structures was adjusted to minimize print time and material wastage. These technical optimizations improved the accuracy and efficiency of preparing 3D printed anatomical models for comminuted fracture preoperative planning, improving opportunities for surgeons to better plan surgical treatment in advance, reducing the likelihood of errors, with the goal of improving surgical outcomes.

## Introduction

Comminuted fractures are a type of fracture that results in bone fragmentation into three or more pieces. These fractures are associated with high-energy trauma and present a complex clinical challenge [1]. Surgical repair of comminuted fractures poses several challenges to the treating surgeon, including the complexity of fracture reduction, the difficulty in maintaining proper alignment of bone fragments, and the risk of surgical errors [2]. These challenges can result in prolonged operative times, increased intraoperative blood loss, and fracture destabilization requiring revision surgery. However, the advent of 3D printing has enabled the fabrication of accurate, patient-specific anatomical models that can aid in surgical planning and potentially improve surgical outcomes to aid in minimizing the impact of several of these challenges [3].

Using medical imaging data, namely computed tomography (CT) scanning, 3D printers can produce highly accurate models that can aid in the visualization of complex fractures, the planning of surgical approaches, and the selection of appropriate medical devices. With the increasing prevalence of point-of-care based facilities, many hospital facilities have the capacity to rapidly produce 3D printed models on request [4, 5]. The use of 3D printing for producing personalized anatomical models is becoming more widespread in orthopedics and trauma surgery, and its impact on improving surgical planning and outcomes is likely to continue to grow in the coming years [6]. There is growing evidence to suggest that the use of 3D printed anatomical models for preoperative planning can lead to significant improvements in surgical outcomes compared to traditional planning methods [6]. Studies assessing the impact of the use of 3D printed anatomical models during preoperative planning in orthopedic contexts have found that their use can reduce surgical time by up to 70 min [7, 8]. Another study found that the use of 3D printed models to perform fixation plate pre-contouring also resulted in shorter surgical time, reduced blood loss and improved postoperative outcomes [9]. Overall, these studies provide strong evidence of the benefits of using 3D printed anatomical models for preoperative planning in orthopedics and trauma surgery.

Producing personalized 3D printed anatomical models is a promising approach to preoperative surgical planning [10], however, this process can be challenging and complex when preparing models representing complex fractures, such as comminuted fractures. Unlike healthy anatomy, comminuted fractures present unique challenges in segmenting bony structures from surrounding tissue and representing these fragments accurately in a physical model [11]. Due to the presence of numerous small, disassociated bone fragments, there is a high chance of misrepresenting fine details and creating artifacts that can lead to inaccurate models and potentially mislead the treating surgeon. The resolution of imaging techniques can further compound this challenge, with some fragments appearing as if they are touching in the scan data, despite being separated in the injury site, due to the partial volume effect. Additionally, when fractures are located within or adjacent to joints, there is an added complexity in ensuring the utility of the 3D model to visualize the fracture from useful angles without being obscured by other anatomical structures. These factors necessitate optimized segmentation protocols and fabrication techniques to produce accurate and useful models for surgical planning [12].

This study therefore presents a series of technical observations and improvements in the production of 3D printed anatomical models for planning the surgical repair of comminuted fractures. To address the challenges of accurately representing complex fractures, this study designed a segmentation protocol to preserve fine details in bone fragments. This was achieved by identifying and separating the individual fragments and removing any artifacts or overlapping structures. In addition, the use of connecting structures, known as "struts", was implemented to maintain the positioning of displaced fragments and ensure their accuracy in the 3D model. The study also utilized magnet positioning to facilitate rapid assembly of multi-part models following fabrication, improving the efficiency of the overall process. Finally, observations regarding the optimization of infill for non-target structures are presented, which can significantly reduce print time and material wastage. Overall, this study provides valuable insights into the technical considerations and advancements in producing personalized 3D printed anatomical models.

## Methods

### Data acquisition

Adult patients referred for pre-procedural computed tomography (CT) to visualize comminuted fractures were included in this study, utilizing retrospectively collected CT data collected at PeaceHealth or Oregon Imaging Center facilities via an IRB-approved collaborative study between the Knight Campus and PeaceHealth hospital network (University of Oregon IRB approval STUDY00000613). A CT scanner (SIEMENS) was used to acquire high-resolution CT scans of the affected lower leg using a 512 × 512 matrix size, 0.31–0.79 mm pixel size and 1–1.5 mm slice thickness. Following CT data acquisition, the DICOM files were exported and anonymized to ensure patient privacy. The anonymized DICOM files were then imported into the software for segmentation and reconstruction of the 3D models.

### Segmentation & Computer Aided Design (CAD)

The DICOM files obtained from the CT scan were imported into Mimics Medical 25.0 (Materialise, Belgium), and the bones of interest were segmented using thresholding, region-growing and manual editing tools. Once the segmentation was complete, the resulting 3D model was refined in 3-matic 17.0 (Materialise, Belgium) by removing any artifacts, adding ‘struts’ to connect floating bone fragments, trimming the model to the desired region of interest, and including specialized strut designs to position magnets, described below. The final 3D model was then exported as an STL file for use in 3D printing using the ‘optimal’ settings. Prior to exporting the STL file, a quality check was performed to ensure that the model was suitable for printing and that there were no errors or gaps in the geometry. This involved visual inspection of the model and the use of automatic tools within the software to detect and repair any issues.

Computer aided design (CAD) of the specialized struts for magnet placement consisted of designing 20 mm length cylinders, 8 mm in diameter, and hollowing a 4.2 mm diameter, 3 mm deep cylinder from the top surface for magnet positioning and suitable tolerance for the addition of cyanoacrylate glue.

CAD of bone-fragment analogues to test the rigidity of different strut lengths and diameters involved designing a 20 × 20 x 20 mm cube adjacent to a 20 × 20 × 3 mm rectangular prism representing the large bone volume and small thin bone fragment respectively. The two objects were then connected with a cylinder of varying length (10 or 30 mm) and diameter (2, 4, 6, 8 or 10 mm).

### 3D printing

STL models were prepared for 3D printing using a material extrusion (MEX) 3D printer (Prusa i3 MK3S + , Prusa Research). STL files were sliced using PrusaSlicer 2.4.2 (Prusa Research) with 0.2 mm layer height, 2 perimeters, 6 top solid layers and 4 bottom solid layers. Infill was set to 20% density ‘grid’ pattern at a 45° fill angle. Support material was automatically generated for overhangs > 50°, consisting of 2 mm spacing rectilinear pattern with 0.2 mm Z contact distance between the model and support and XY separation set to 60% of the model perimeter thickness. Physical models were fabricated using PLA filament of various colors (eSUN PLA + 1.75 mm) and support structures were removed during manual post-processing to result in the final products.

### Analysis and Clinical Feedback

Images and videos of handling the 3D printed models were acquired with a smartphone camera (Samsung S20) and digital cropping performed to remove extraneous background. Surgeons were provided the models prior to and following surgery and asked to comment on the relative importance of visualization of various aspects of the anatomy in the context of the injury locations, responding via short answers and analyzed using thematic analysis.

## Results & discussion

### Cortical bone segmentation enables high-precision fracture visibility

**Fig. 1:**
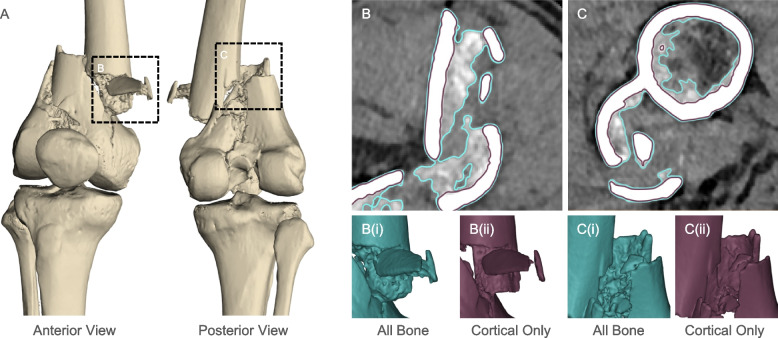
Comparison of segmenting entire bone volume versus cortical bone only for representing comminuted fracture fragments. **A** Overview of a comminuted femoral fracture case and two identified regions of interest. **B**, **C** DICOM images with overlayed outlines of the two STL files generated from (i) segmentation of all bone structures using thresholding (teal) versus (ii) segmentation of the cortical bone only (maroon)

To investigate the ability to visualize bone fragments within a comminuted fracture, a case study involving a patient who had suffered from a comminuted fracture of the right distal femur was identified. Here, the entire bone volume was initially segmented from the CT images to provide an overview of the injury and its proximity to the knee joint (Fig. 1A). To achieve segmentation of the entire bone volume, routine segmentation was performed by firstly applying a threshold using the BoneCT default limits provided by the Mimics software (Materialise). Subsequently the Multiple Slice Edit tool was used to manually add to the segmentation mask using interpolation between multiple slices to restore the morphology of underexposed bone regions including particularly thin structures that were not captured in the original mask. For specific cortical bone segmentation, narrower threshold limits were applied to isolate the higher intensity pixels corresponding to denser bone. Using this as the template, more manual edits were required to re-include fine structures and other anomalies in the segmentation mask. In the context of reconstructing complex fractures such as in this case study, segmentation of the cortical bone, as opposed to the entire bone volume, proved to be an effective technique for resolving the most important fracture components. This approach provided a more accurate representation of the morphology of fractured bone fragments of greatest potential for use in reconstruction (Fig. 1B). By precisely resolving the cortical bone structures, better visibility into cortical or cartilage-interfacing surfaces that have been displaced and embedded into the center of the bone fracture can be seen (Fig. 1C).

In a typical 3D printed model printed using a single material, it can be difficult to distinguish between small bone fragments and trabecular bone since they are both printed from the same material and may be similar in scale in the final representation. Given the 0.5 mm resolution of CT scanning and sub-0.5 mm feature size of individual trabeculae [13], it is beyond the intended application of this imaging, modelling and fabrication modality to precisely represent trabecular bone structures accurately. Other modalities such as micro-CT have been explored in research contexts to specifically resolve such structures [14] or use variable infill to approximate trabecular structures [15]. Using clinically relevant CT scan resolutions, resulting attempts to include trabecular bone within the segmentation and 3D model yield overly smoothed and low-resolution appropriations of the trabecular network that in some instances can be indistinguishable from sub-centimeter scale fragments of cortical bone. This misappropriation of trabecular bone is visualized both in the smoothed teal outline in Fig. 1B surrounding the trabecular bone attached to the vertical cortical bone fragment, as well as comparing the smooth morphology of both a small cortical bone component embedded with the trabecular network of the distal femoral components in Fig. 1C. Resolving the cortical bone structures in isolation during the segmentation processes helped overcome this challenge by providing an accurate representation of the most important fracture components to inform surgical repair. Whilst in other contexts there may be clinical utility to visualizing the trabecular bone morphology, in this case, preference was given to resolving the cortical bone components since in periarticular fractures, the cortical bone surface is vital to judge the reduction of the fracture.

### Connecting strut positioning retains positioning of displaced fragments

**Fig. 2:**
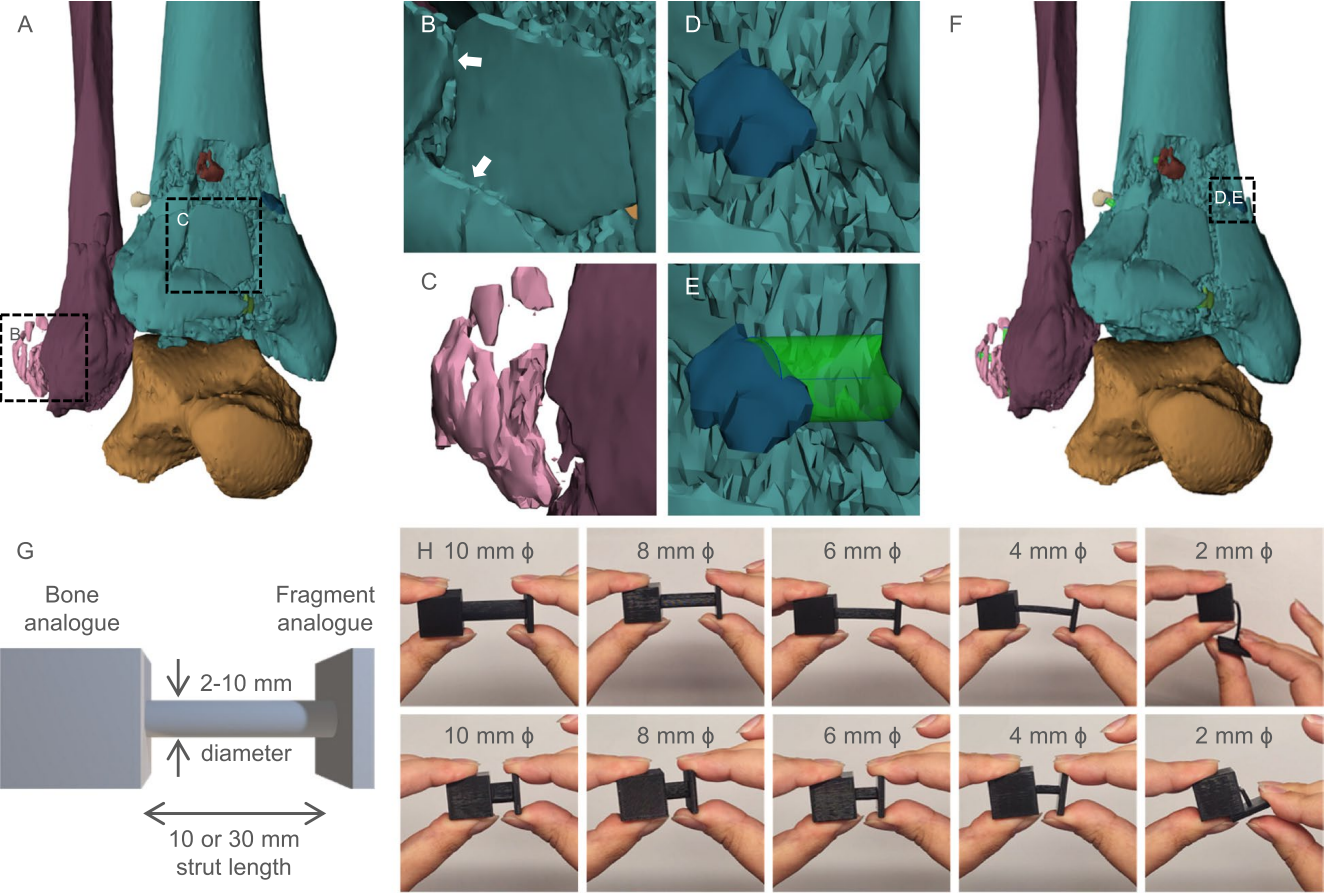
Identification of floating fragments and design of connecting struts to maintain their position. **A** 3D modelling segmentation of tibia (teal), fibula (maroon) and talus (orange) bones, with small floating components visualized in other colors. Identification of (**B**) thinly connected and (**C**) completely disconnected (floating) bone fragments. **D**, **E** Design of cylindrical connecting struts to stabilize the floating components with respect to the larger bone model. **F** Final digital model with connecting struts added to all floating components. **G** Schematic of a simulated bone and fragment component and connecting strut of varying diameter. **H** Handling of the 3D printed simulated components revealed the likelihood of component breakage

In instances of comminuted fractures, the prevalence of disconnected or ‘floating’ fragments that do not interface with adjacent bones is substantial. To illustrate this, a Pilon fracture case (right leg) was identified, featuring comminuted tibial and fibular fractures and several floating or thinly connected bone fragments (Fig. 2A). These floating components, particularly those derived from cartilaginous joint surfaces are of critical importance during fracture repair, as they will often be salvaged to reconstruct the joint surface. However, conserving their relative position within the injury site is a challenge when producing a physical replica. This also applies to components with thinly connecting fragments, often due to extremely close positioning below the resolution of the imaging modality (Fig. 2B, C). To ensure that a high degree of accuracy is maintained in representing the location of floating fragments, connecting struts are proposed as an identifiable, aesthetically non-organic structural additions to the 3D printed models to physically connect the floating components. CAD software platforms such as 3-matic have an inbuilt option for adding connecting struts between components. This tool, in combination with manual design and Boolean addition of cylindrical objects was used to connect small fracture fragments to the larger bone component (Fig. 2D-F).

Two key design criteria are recommended for placing the connecting struts. First, strategically selecting locations where the strut could interface with the components but did not substantially impede visualization of fracture margins was critical. Angling of the struts to interface with the bone component within a region of exposed trabecular bone or internal surface of cortical bone preferred since the specific architecture of the internal structures was of low interest to the surgeons, based on qualitative feedback received. According to the feedback, impeding visibility of these regions did not compromise the utility of the model (Fig. 2E). Secondly, the diameter of the strut had to be large enough to provide sufficient rigidity to the component to withstand removal from the print bed, removal of support material, and handling by the surgeons, whilst not impacting visual access to the fragment morphology. To provide a guide to representative strut thicknesses and rigidity during handling, simulated bone-fragment components, positioned 10 mm and 30 mm apart and with struts of 2, 4, 6, 8 and 10 mm diameter were designed in CAD and 3D printed using the same filament, printer, and print conditions as the physical models (Fig. 2G).

During handling, the simulated fragment (Fig. 2G) could be bent to angles of approximately 10 degrees and 90 degrees (maximum bending) for struts 4 mm and 2 mm in diameter respectively, at a strut length of 30 mm (Fig. 2H). However, repeated bending of the 4 mm strut could only be performed 5 times before the strut broke at the intersection with the simulated bone. Importantly, these simulated samples were printed with the strut oriented parallel to the print bed. Since for MEX in particular, mechanical properties are strongly informed by part orientation due to anisotropy in the fabrication of successive layers, a test sample with the strut oriented perpendicular to the print bed was printed and exhibited even weaker handling rigidity and struts of either length, 4 mm or 2 mm, immediately snapped upon handling/bending. These representative tests suggest that whilst struts 4 mm or larger in diameter are likely to be acceptable using the filament and print slicing settings applied in this study, struts of 2 mm diameter may only be acceptable when connecting distances < 10 mm and oriented parallel or close-to-parallel to the print bed. A larger number of perimeter lines to increase the wall thickness or denser infill pattern would strongly influence the mechanical strength of printed struts and should be optimized accordingly to print both the struts and entire anatomical model using consistent parameters.

In general, the smallest strut thickness is preferred to minimize impact on visual access to the anatomy whilst providing sufficient rigidity based on the displacement distance of the fragment to the adjoining location on the main bone component. In the event that the connection tool output did not satisfy these two criteria, cylinders were manually generated and combined with the fragment and main bone component via the Boolean union operation.

Ideally, additions to the 3D printed model would be fabricated in a different color to clearly delineate between anatomy and fabrication constructs, however with single-material fabrication modalities such as traditional MEX used in this study, this is not achievable. Other printers such as multi-material extrusion printers or multi jet fusion full-color printers may offer an added degree of complexity and coloring to the models for clearly distinguishing between native and digitally added morphologies, as well as allowing for visibility of key structural variance in tissues, such as cartilage-interfacing surfaces [16], to improve identification on the model and inform reconstructive procedures.

### Magnets facilitate reconstruction of multiple bones

**Fig. 3:**
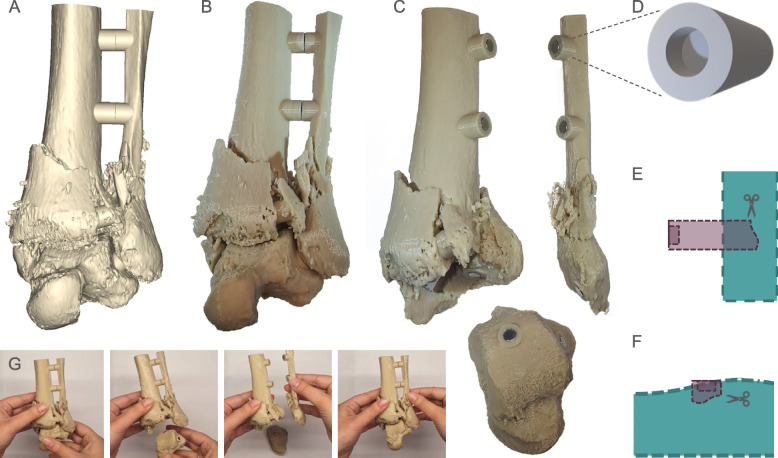
Application of magnetic connection struts to create separable models. **A** CAD model of the comminuted distal tibial and fibular fracture and (**B**) 3D printed model. **C** Application of magnetic connector struts to enable separation and reconnection of adjacent bones. **D** STL model of the generalized magnetic holder connector that was intersected with either (**E**) long bones or (**F**) irregular bones and trimmed within the margins of the bone. **G** Handling of the model demonstrating detachment of magnetic parts and reassembly

As an alternative to rigid connecting struts between disconnected features of the fracture models, magnets have been proposed to enable removal and reconnection of separable or split parts and thus increase visibility of anatomy normally obscured by adjacent features [4, 17, 18]. In this context, fracture models involving multiple bones at joints benefit strongly from the use of magnets embedded within the anatomy or connecting struts to demonstrate the positioning of bones and proximity of fracture fragments with respect to adjacent bones [18]. To exemplify the utility of magnetic connections and precise conservation of bone positioning, a second Pilon fracture case involving a comminuted distal tibial and fibular fracture of the left leg was identified (Fig. 3A-C). Per previously described methods, a 3D model of the anatomy, including the adjacent uninjured talus was created in Mimics and exported to 3-matic. Here, an STL model of a cylindrical connector strut with an insert for a 4 mm diameter magnet (Fig. 3D) was imported into 3-matic and replicated several times. Following alignment of pairs of the magnet holder to face each other to enable the magnets to connect, the cylindrical structures were positioned between bones of the model such as between the tibia and fibula (Fig. 3A-C). Extra connector length was trimmed within the margins of the intersecting long bone (Fig. 3E) or embedded within the planar surface of the irregular bone (Fig. 3F). Once assembled, the part could be easily handled, with independent parts readily detached from each other or reassembled to enable close inspection of the fracture at the articulating surface between the tibia and fibula (Fig. 3G).

### Infill design for non-target structures

In the context of 3D models of long bones, segmentation of bones often yields hollow structures with complex internal architecture due to the presence of the medullary cavity. The interior surface of the bone therefore contains a high prevalence of small features corresponding to the segmentation of highly porous trabecular bone lining the cavity. In regions of the 3D model outside the target anatomy of interest, such as a fracture, it may not be strategic to print this fine detail on the interior surface of non-target structures accurately since it may not be visible within the interior of the model and does not interfere with representing the specific anatomy of interest. This study proposes prioritizing faster printing speeds and less risk of introducing print errors in non-target regions of the 3D model by relying on the slicer generated infill patterning rather than attempting to resolve the fine detail of the interior bone surfaces and use of extensive support structures to print overhanging or hollow internal features.

**Fig. 4:**
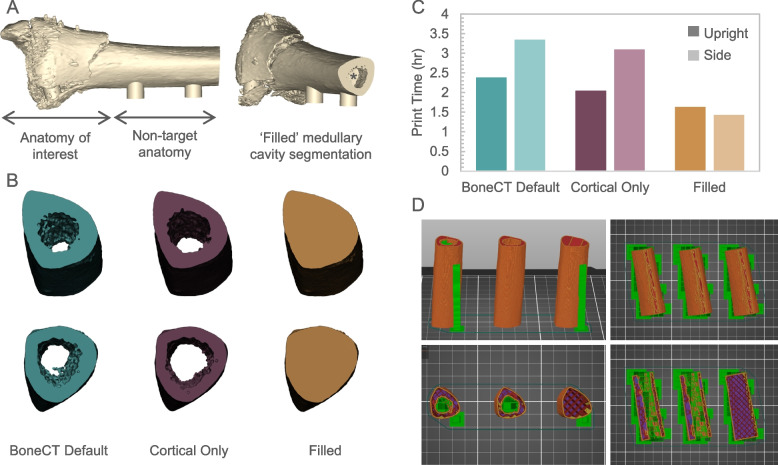
Comminuted distal tibial fracture model to illustrate use of infill versus supports for fabrication of non-target internal anatomy. **A** Digital 3D model of the tibial fracture case, including the fracture region and additional tibial shaft. (*) Asterisk indicates manually filled cavity. **B** Digital 3D models of the 70 mm tibial shaft segmented using the BoneCT Default threshold, threshold of cortical bone only and manually filled medullary cavity. **C** Comparison of the print time calculated by the slicing software for each tibial shaft model when oriented upright or on its side relative to the print bed. **D** Visual representation of the slicer output for each model in each orientation, showing the generated perimeter path (orange), support structures (green), infill (red), bottom solid layers (purple), and overhangs (blue)

To illustrate this proposition, the case study introduced earlier involving a comminuted distal tibial and fibular fracture of the left leg was selected, where a length of the tibial shaft proximal to the injury was included in the model to enable planning of periarticular plate placement (Fig. 4A). Following segmentation via the default BoneCT threshold limits, it was observed that the interior surface of the uninjured tibial shaft segment featured a highly intricate network of trabeculae, the individual bony structures comprising trabecular or spongy bone (called ‘BoneCT default’ segmentation). Two alternative approaches were proposed: segmentation of the cortical structure only by selecting a narrower threshold window to eliminate lower density bony structures from the segmentation mask (called ‘Cortical Only’ segmentation), or by using the Multiple Slide Edit tool to manually include the entire medullary cavity in the segmentation mask between a distal limit > 5 mm superior to the fracture and the proximal limit of the scan data (called ‘Filled’) (Fig. 4B). By comparing the slicing results of these 70 mm lengths of the tibial shaft segmented via each of these three methods, the opportunity to reduce print time and improve print accuracy was compared.

When oriented upright on the print bed, the BoneCT Default model took 2 h 23 min to print, with extensive and largely inaccessible support structures required within the center of the construct (green) due to the presence of intricate trabecular structures surrounding the medullary cavity, despite the majority of the bone structure being self-supporting when positioned vertically (orange) (Fig. 4C, D). A 14% reduction in print time and 8% reduction in filament usage was achieved using the Cortical Only segmentation method, whilst a 31% reduction in print time and 23% reduction in filament usage was achieved using the ‘Filled’ method that relies on the slicer-generated support structure to fill the medullary cavity. This was further exacerbated when orienting the bone on its side on the print bed. The use of the ‘Filled’ segmentation method led to a 57% reduction in print time, from 3 h 21 min to 1 h 26 min, and 33% reduction in filament usage compared to the BoneCT Default segmentation model (Fig. 4C, D). By filling the cavity during segmentation, the slicing process was reliant on programmed 20% infill (red) rather than challenging small feature fabrication with overhanging structures requiring extensive support material to achieve the same exterior morphology.

This method eliminates redundancy in fabricating high precision structures with limited clinical utility. Here, we circumvent the need to intricately print multi-walled structures within the cavity that are not readily visible, cannot be accurately fabricated (see previous section), and are outside the region of interest for the 3D printed model. Thus, more rapid and less wasteful model fabrication can be achieved. There exists an opportunity in the slicer to locally remove the internal perimeters from the medullary cavity surface and design infill within the cavity in the slicing software. However, this is not recommended due to the inability to accurately identify structures of interest (such as fracture fragments) once the part is rendered as an STL file. It is recommended to perform any identification of target and non-target structures, and design where infill will be utilized, during the segmentation stage such that localized variations to the segmentation mask from the native geometry can be tightly controlled and visible overlaid on the DICOM images.

In addition to the quantifiable metrics indicating more efficient fabrication, the use of infill rather than complex geometry coupled with supports drastically reduces the risk of print error and failure. The infill pattern is a highly linear, reproducible pattern that can be accurately fabricated with very low risk of error compared to attempting to resolve intricate trabecular structures, often below the resolution of the fabrication technique, and use of extensive support structures to reinforce and stabilize the part during fabrication (Fig. 4D). Once an error in the fabrication occurs, there is a high risk of the entire part failing due to the propagation of errors in subsequent layers. Preference to utilize infill rather than printing redundant complex geometry and supports strongly reduces the risk of fabrication failure, production of waste and extended lead times which are of critical importance to the successful operation of a point-of-care 3D printing facility [19].

In its final representation, a filled medullary cavity was incorporated into the tibial model, allowing a 5 mm portion of the medullary cavity to be viewed at the proximal end of the model to enable visualization of cortical bone thickness to inform reaming and intermedullary nail placement, and filled to a depth of 5 mm superior to the fracture as to not interfere with accurate representation of the fracture anatomy (Fig. 4A*).

The technical improvements to the workflow for preparing 3D printed anatomical models for fracture intervention planning presented in this study contribute directly to enhancing the feasibility for implementation in a clinical setting. The reduction in print time achieved, coupled with the reduction in risk of print failure, are aligned with the clinical requirement to produce the 3D printed model and allow sufficient time for clinical review prior to surgery. In the case of major trauma, clinical practice guidelines underscore the importance of timely intervention. For example, in femoral shaft fracture intervention, delayed fixation is defined as > 24 h and was found to lead to increased risk of pulmonary embolism and longer hospital stays [20]. Therefore, the 3D printing workflow should be designed for a "same day" turn-around, which this study has shown to be feasible with technical enhancements to the segmentation process and reduction in printing time. By facilitating a rapid production of precise anatomical models for preoperative planning, the proposed workflow harmonizes with the clinical practice guidelines, enabling surgeons to visualize and simulate the complex fracture reduction preoperatively.

There is significant potential for further research into leveraging different 3D printing techniques, such as stereolithography or material jetting printing technology [19], to optimize anatomical model production, as many of the modelling and fabrication steps presented in this study are specific to addressing challenges in fabricating the models using MEX. Using other fabrication technologies, alternative strategies may be implemented to optimize component strengths, material usage and printing efficiency. Further research using these technologies may develop innovative approaches to reduce print failures, optimize print times, and potentially achieve superior results in model accuracy and structural integrity across a broad range of fabrication modalities.

## Conclusion

This study has presented and validated the use of four specific 3D modelling and 3D printing tools to improve the design and fabrication of anatomical models for comminuted fracture preoperative planning. The optimized segmentation protocol, inclusion of connecting struts, use of magnets, and optimization of infill for non-target structures contribute to more accurate and efficient preparation of 3D printed anatomical models specifically in the context of comminuted bone fracture modelling. However, learnings from these modelling strategies have applications beyond this specific clinical context. The use of these technical optimizations provides benefits for surgeons to visualize and simulate fracture reduction processes before surgery, reducing the likelihood of errors, and improving surgical outcomes. The inclusion of magnets allowed for the creation of separable models of multiple bones, providing a better understanding of the proximity of fracture fragments and the relative positioning of bones, which can assist in planning surgical approaches. By minimizing material wastage and print time, the optimized fabrication process presented in this study not only improves the efficiency and accuracy of 3D printed anatomical models but also contributes to reducing the environmental impact associated with 3D printing technology, aligning with a growing sustainability agenda in healthcare. These technical advancements in 3D printed anatomical models provide valuable tools for preoperative planning of comminuted fracture repair, improving opportunities for surgeons to better plan surgical treatment in advance and potentially translating to improved patient outcomes.

## Data Availability

The datasets analyzed during the current study are not publicly available but are available from the corresponding author on reasonable request.
